
JOURNAL INFORMATION
==============================
NLM Title Abbreviation: Biospektrum (Heidelb)
Journal ID: BIOspektrum (Heidelb.)
NLM Journal ID: 9615685

Biospektrum

ISSN: 0947-0867
EISSN: 1868-6249

ARTICLE INFORMATION
==============================
PMCID: PMC7474486
PMID: 32921925
DOI: 10.1007/s12268-020-1431-1
Article ID: 1431
Article version: 1
Subjects: Wissenschaft Special

Etablierung der PCR-basierten SARS-CoV-2-Testung im Hochdurchsatz

Klussmeier Anja klussmeier@dkms-lab.de

Behrens Geoffrey A.
Lange Vinzenz
DKMS Life Science Lab GmbH, St. Petersburger Straße 2, D-01069 Dresden, Deutschland
Electronic publication date: 2020 Sep 5
Print publication date: 2020
Volume: 26
Issue: 5
First page: 500
Last page: 503
Copyright: © Der/die Autor(en) 2020
License: Open Access Dieser Artikel wird unter der Creative Commons Namensnennung 4.0 International Lizenz veröffentlicht, welche die Nutzung, Vervielfältigung, Bearbeitung, Verbreitung und Wiedergabe in jeglichem Medium und Format erlaubt, sofern Sie den/die ursprünglichen Autor(en) und die Quelle ordnungsgemäß nennen, einen Link zur Creative Commons Lizenz beifügen und angeben, ob Änderungen vorgenommen wurden.
Die in diesem Artikel enthaltenen Bilder und sonstiges Drittmaterial unterliegen ebenfalls der genannten Creative Commons Lizenz, sofern sich aus der Abbildungslegende nichts anderes ergibt. Sofern das betreffende Material nicht unter der genannten Creative Commons Lizenz steht und die betreffende Handlung nicht nach gesetzlichen Vorschriften erlaubt ist, ist für die oben aufgeführten Weiterverwendungen des Materials die Einwilligung des jeweiligen Rechteinhabers einzuholen.
Weitere Details zur Lizenz entnehmen Sie bitte der Lizenzinformation auf http://creativecommons.org/licenses/by/4.0/deed.de.
License URL: https://creativecommons.org/licenses/by/4.0/

issue-copyright-statement: © Springer-Verlag GmbH Deutschland, ein Teil von Springer Nature 2020

==============================
Timely identification and isolation of affected individuals is one of the most important factors to control spreading of the newly emerged SARS-CoV-2. Consequently, it is crucial to maintain sufficient sample capacities in diagnostic laboratories. Here, we present a high-through-put approach for PCR-based SARS-CoV-2 testing. The implementation of sample pooling reduces costs and workload, especially in times with low population prevalence.

■ Im Dezember 2019 wurde das neuartige Coronavirus SARS-CoV-2, Auslöser der Erkrankung Covid-19, im chinesischen Wuhan erstmalig beschrieben [1]. Seitdem stellt es die staatlichen Gesundheitssysteme weltweit vor große Herausforderungen. Nach ersten lokal begrenzten Geschehen im Januar 2020 erreichte die Anzahl diagnostizierter SARS-CoV-2-Infektionen in Deutschland Mitte März 2020 einen Höhepunkt.

Eine entscheidende Maßnahme zur Eindämmung des SARS-CoV-2-Infektionsgeschehens ist die rechtzeitige Diagnose von Einzelfällen. Auf diese Weise können mögliche Kontaktpersonen identifiziert, Quarantänemaßnahmen verhängt und Infektionsketten unterbrochen werden. Ende März 2020 war daher abzusehen, dass bei einem stark steigendem Infektionsgeschehen, wie es zu diesem Zeitpunkt in Italien oder Spanien beobachtet werden konnte, hohe Laborkapazitäten für die Diagnostik von SARS-CoV-2 in Deutschland benötigt werden würden.

Das DKMS Life Science Lab ist das weltweit größte Labor für die HLA-Genotypisierung von Personen, die sich für eine Stammzellspende registrieren möchten. Pro Werktag werden hier rund 5.000 Proben im Hochdurchsatz bearbeitet: von der Annahme der Abstrichtupfer, über die DNA-Isolation, PCR und Sequenzierung bis hin zur Datenanalyse [2, 3]. Für diese Prozesse steht eine umfassende Labor-Infrastruktur und -Automation zur Verfügung. Jeder Arbeitsschritt wird durch ein Laborinformations-Management-System (LIMS) abgebildet, welches an die speziellen Anforderungen des Labors angepasst ist. Auf diese Weise können Proben automatisiert in die für sie geplanten Arbeitsschritte geleitet werden. Dies stellt nicht nur einen schnellen Laborablauf sicher, sondern ermöglicht mit einer kontinuierlichen Überwachung des Gesamtprozesses auch eine umfassende Qualitätssicherung. Die LIMS-Datenbank ist dafür mit einer Daten-Visualisierungssoftware (Tableau Server) verbunden, die in Echtzeit übersichtliche Grafiken erstellt, mit denen Prozessabweichungen zeitnah identifiziert werden können. Um diese komplexen Automations- und IT-Strukturen schnell und flexibel auf neue Projekte anpassen zu können, erfolgt die Umsetzung durch eigene Mitarbeiter. Dies ermöglicht eine weitgehende Unabhängigkeit, die nicht von Zeitplänen externer Dienstleister bestimmt wird. In den letzten Jahren haben wir diese Kompetenzen bereits Projektpartnern zur Verfügung gestellt, die hohe Probenaufkommen bewältigen mussten. Ein solches Projekt beinhaltete z. B. die DNA-Isolation von eingesandten Abstrichtupfern, die Normalisierung der DNA sowie deren Reformatierung auf 384-Well-Platten für mehrere zehntausend Proben. Aufgrund dieser besonderen Fähigkeiten unseres Labors haben wir im März 2020 angesichts der Krise begonnen, die SARS-CoV-2-Diagnostik im Hochdurchsatz aufzubauen.

## Laborprozess

Das Standardverfahren für die SARS-CoV-2-Diagnostik ist die Isolation von Virus-RNA aus einem Rachenabstrich mit anschließender RT-PCR [4]. Dafür stand schon im März 2020 eine Auswahl validierter Kits verschiedener Hersteller zur Verfügung. Unser Ziel war es, eines dieser Kits in einen Laborprozess mit möglichst hohem Probendurchsatz zu integrieren (**Tab. 1**). Als Flaschenhals wurde eine maximale Kapazität von 8.000 RNA-Isolationen/Arbeitstag identifiziert. Um die Kapazität darüber hinaus zu steigern, haben wir einen Prozess konzipiert und etabliert, der die Vereinigung von sechs Proben vor der RNA-Isolation erlaubt. Dieses Verfahren ermöglicht es, bis zu 10.000 SARS-CoV-2-Tests pro Tag parallel zu unserer HLA-Genotypisierung durchzuführen.

Der SARS-CoV-2-Prozess beginnt mit der Einsortierung der Abstrichtupfer in 96-Well-Platten (**Abb. 1**). Die Barcodes der Proben werden dabei registriert und mit ihrer Position und Plattennummer im LIMS verknüpft. Durch die Zugabe von Lysepuffer erfolgt die Inaktivierung des infektiösen Materials, sodass die Proben danach außerhalb der Sicherheitswerkbank automatisiert bearbeitet werden können. Für den Pooling-Ablauf vereinigt ein Pipettierroboter nun einen Anteil von jeweils sechs Lysaten. Auf diese Weise kann die RNA von bis zu 540 Proben auf einer 96-Well-Platte isoliert werden. Die verbleibenden Reste der Original-Lysate werden für eine eventuell notwendige Einzelanalyse oder Wiederholung zurückgestellt. Aus vier 96-Well-RNA-Isolationsplatten bildet ein weiterer Roboter schließlich eine 384-Well-RT-PCR-Platte.

Nach Durchlaufen der RT-PCR-Reaktion werden die Rohdaten durch einen Labormitarbeiter qualitätsgeprüft und anschließend in das LIMS überführt. Dort werden die Ct-Werte der RT-PCR entsprechend der Vorgaben des Kits automatisch in die Ergebnisse SARS-CoV-2-positiv, SARS-CoV-2-negativ oder SARS-CoV-2-grenzwertig übersetzt und über einen eingepflegten Algorithmus verschiedene Prozesse ausgelöst: Alle SARS-

Hier steht eine Anzeige.

Springer

**Tab. 1:** Laborautomation und Kits, die für den SARS-CoV-2-Prozess verwendet werden. Nicht aufgeführt sind diverse Kleingeräte sowie selbst entwickelte Softwarelösungen.

| Schritt | Laborautomation/Kit | Hersteller | Anzahl |
|---|---|---|---|
| **Probenmanagement** | BaseSpace Clarity LIMS | Illumina | |
| **Pooling** | Freedom EVO | Tecan | 2 |
| **RNA-Isolation** | chemagic MSM I | PerkinElmer | 3 |
| | chemagic 360 | PerkinElmer | 1 |
| | Carousel-LPX-440 | LiCONiC Instruments | 3 |
| | Biomek-NXP / -NX-S8 | Beckman Coulter | 3 |
| | chemagic Viral DNA/ RNA 300 Kit H96 | PerkinElmer | |
| **RT-PCR-Ansatz** | Freedom EVO | Tecan | 2 |
| | SARS-CoV-2 real-time RT-PCR assay CE-IVD | PerkinElmer | |
| **RT-PCR** | QuantStudio 7 Pro | ThermoFisher Scientific | 3 |

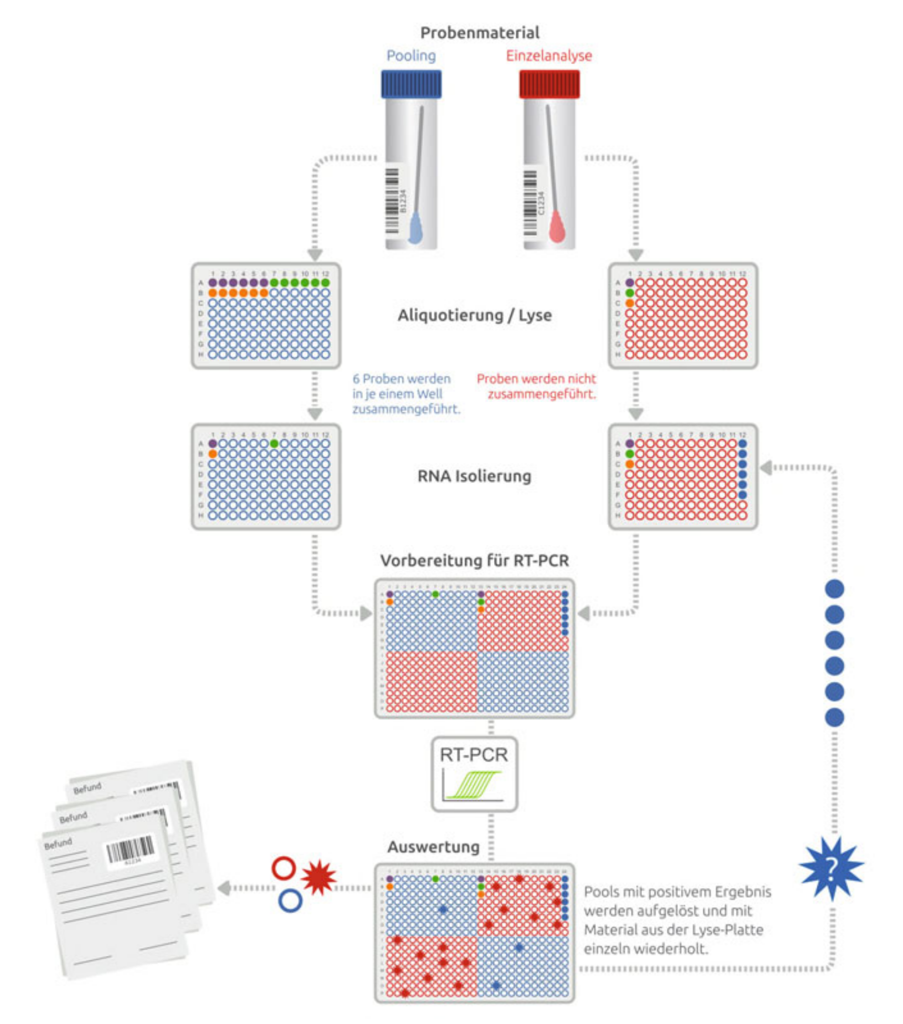

▲ **Abb. 1:** Laborablauf SARS-CoV-2-Diagnostik. Im Pooling-Ablauf werden die Lysate von 6 Proben vereinigt. Nach der RNA-Isolation wird aus vier 96-Well-Platten eine 384-Well-RT-PCR-Platte generiert. Bei negativen Pool-Ergebnissen können unmittelbar Befunde für alle Proben des Pools erstellt werden. Ist ein Pool SARS-CoV-2-positiv, durchlaufen die 6 Proben eine Einzelanalyse ab RNA-Isolation. Bei Proben mit hoher Infektionswahrscheinlichkeit oder Dringlichkeit kann die Einzelanalyse auch als eigenständiger Prozess verwendet werden.

CoV-2-negativen Proben werden akzeptiert und zur medizinischen Freigabe gesendet. Dies gilt auch für SARS-CoV-2-negative Pools, für die das Ergebnis automatisch auf alle darin enthaltenen Proben übertragen wird. SARS-CoV-2-positive Ergebnisse werden vom LIMS in zwei Richtungen gelenkt: Einzelproben gehen in die medizinische Freigabe. Bei Pools bedeutet ein SARS-CoV-2-positives oder -grenzwertiges Ergebnis jedoch, dass eine oder mehrere Proben im Pool das Virus tragen könnten. Daher werden alle im Pool enthaltenen Proben an den Start der Einzelanalyse zurückgesetzt, wodurch eine weitere RNA-Isolation und RT-PCR aus dem ursprünglichen Lysat eingeleitet wird. Dafür erstellt das LIMS eine Arbeitsliste, anhand derer ein Labormitarbeiter den Pipettierroboter mit den entsprechenden Original-Lysatplatten belädt. Vom LIMS gesteuert, pipettiert der Roboter anschließend die entsprechenden Probenlysate auf die Platte für eine erneute, in diesem Fall jedoch ungepoolte, RNA-Isolation.

Die durch das LIMS ermittelten finalen Ergebnisse werden dem für die Freigabe der Befunde zuständigen medizinischen Personal schließlich in einer grafischen Oberfläche (Tableau Server) zur Verfügung gestellt. An dieser Stelle sind außerdem alle Kommentare sichtbar, mit denen die Labormitarbeiter potenziell aufgetretene Abweichungen zum Standardprozess dokumentieren. Auf diese Weise ist sichergestellt, dass nur valide Ergebnisse ausgegeben werden. Die Erzeugung des Befunddokuments für den Einsender und das zuständige Gesundheitsamt erfolgt nach medizinischer Freigabe ebenfalls automatisch.

Die beschriebene, feingliedrige Prozesssteuerung durch das LIMS ermöglicht neben einer effektiven Kontrollfunktion auch eine hohe Bearbeitungsgeschwindigkeit der Proben ohne personalbedingte Pausen. Dadurch kann der größte Nachteil eines Pooling-Verfahrens, die doppelte Bearbeitung positiver Proben mit entsprechendem zeitlichen Aufwand, auf das notwendige Minimum reduziert werden. Damit wird sichergestellt, dass die Befunde infizierter Personen trotz Pooling-Verfahren zeitnah übermittelt werden können.

## Validierung

Alle Laborprozesse wurden mit AccuPlex™-SARS-CoV-2-Referenzmaterial (seracare) validiert. Für die Einzelanalyse wurden 12 unabhängige Verdünnungsreihen in 32 RT-PCR-Reaktionen mit drei verschieden RT-

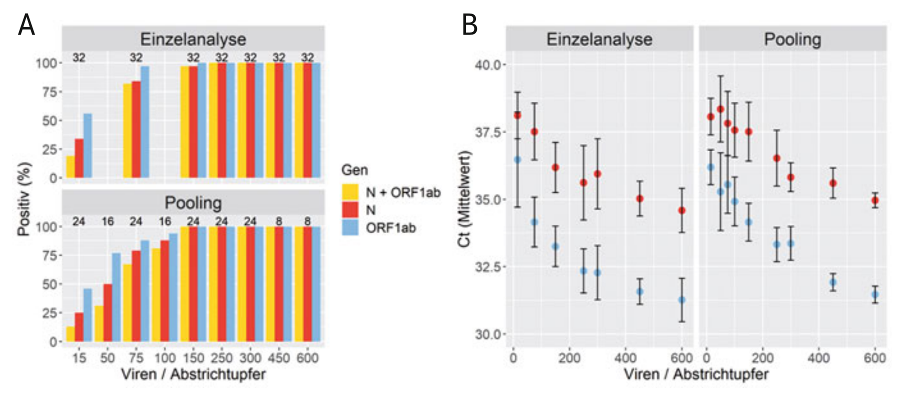

▲ **Abb. 2:** Bestimmung des Detektionslimits. Definierte Mengen eines SARS-CoV-2-Referenzmaterials wurden in Replikaten mit dem Pooling- oder Einzelanalyse-Ablauf analysiert. **A**, Anteil positiver RT-PCR-Ergebnisse für beide bzw. die einzelnen SARS-CoV-2-Gene pro Verdünnungsstufe (n=8-32). **B**, Ct-Werte für das *N*- (rot) bzw. *ORF1ab-Gen* (blau) bei positivem RT-PCR-Ergebnis.

PCR-Geräten analysiert. Für die gepoolte Analyse wurden 12 unabhängige Verdünnungsreihen in 8 bis 24 RT-PCR-Reaktionen durchgeführt (**Abb. 2**). Das *ORF1ab*-Gen des SARS-CoV-2-Virus zeigte sich dabei mit niedrigeren Ct-Werten sensitiver als das *N*-Gen. Eine Probit-Analyse ergab für die Einzelanalyse ein 95 %-Detektionslimit von 130 Viren pro Abstrichtupfer. Nach Durchlaufen unseres Laborprozesses entspricht dies 5,6 RNA-Kopien in einer 20-µl-RT-PCR-Reaktion. Für gepoolte Proben lag das ermittelte 95 %-Detektionslimit bei 251 Viren pro Abstrichtupfer (5,4 RNA-Kopien in der RT-PCR-Reaktion). Dies zeigt, dass durch die initiale Vereinigung von Proben nur ein geringes Maß an Sensitivität eingebüßt wird, welche immer noch im Rahmen verfügbarer zertifizierter Testsysteme liegt [5]. Darüber hinaus ist bei einer durchschnittlichen Viruslast um die $10^5$ Kopien/ml Lysat nur ein sehr geringer Anteil der Patientenproben im grenzwertigen Bereich zu erwarten [6, 7].

## Fazit

Der hier beschriebene Laborprozess zur Diagnostik von akuten SARS-CoV-2-Infektionen wurde in einem Zeitraum von etwa drei Monaten geplant, umgesetzt und validiert. Ein großer Teil dieser Zeit wurde in eine benutzerfreundliche und weitestgehend automatische Funktionalität der Probensteuerung, -überwachung und -befundung investiert. Dies ermöglicht insbesondere die initiale Vereinigung von Proben mit einer automatisch ausgelösten Einzelanalyse positiver Pools. Bei Anpassung unserer betrieblichen Abläufe (Drei-Schicht-Betrieb, keine parallele Bearbeitung von HLA-Spenderproben) könnte damit eine maximale Kapazität von bis zu 25.000 SARS-CoV-2-Proben/Arbeitstag erreicht werden. Seine hohe Zeit- und Kosteneffizienz beweist das eingesetzte Verfahren vor allem bei einer niedrigen Prävalenz in der Testgruppe [8]. Darunter fallen z. B. Reihenuntersuchungen von asymptomatischen Personen, die zum gezielten Schutz von Risikobereichen (Gesundheitswesen, besonders gefährdete Betriebe) eingesetzt werden.

Mit unserem etablierten Prozess bilden wir als Speziallabor, welches normalerweise keine Infektionsdiagnostik durchführt, nun eine ergänzende Ressource, die bei epidemiologischer Notwendigkeit aktiviert werden kann.

## Danksagung

Die zeitnahe Implementierung einer SARS-CoV-2-Diagnostik parallel zum laufenden Tagesgeschäft stellte unser Labor in den letzten Wochen vor so manche Herausforderung. Diese wäre ohne den hohen Einsatz aller Mitarbeiter nicht zu leisten gewesen. Ihnen gilt daher unser ganz besonderer Dank. ■


Funding Open Access funding provided by DKMS Life Science GmbH, Dresden.

Autoren

Anja Klußmeier

2001–2006 Studium der Humanbiologie an der Universität Marburg. 2011 Promotion in Biochemie, FU & Charité Universitätsmedizin Berlin. 2016–2018 Senior Scientist und seit 2018 Head of Research & Development der DKMS Life Science Lab GmbH in Dresden.

Geoffrey A. Behrens

2005–2010 Studium der Biochemie an der Universität Greifswald. 2014 Promotion in Biochemie, Universität Greifswald. 2014–2016 Postdoktorand an der Rijksuniversiteit Groningen, Niederlande. 2016–2019 Spezialist für Labortechnik und Automation und seit 2019 Business Development Manager bei DKMS Life Science Lab GmbH in Dresden.

Vinzenz Lange

1994–2000 Studium der Biochemie an der Universität Regensburg. 2006 Promotion am Max-Planck-Institut für Molekulare Zellbiologie und Genetik, Dresden. 2006–2008 Postdoktorand am Institut für Molekulare Systembiologie ETH Zürich, Schweiz. 2009–2015 Abteilungsleiter Labortechnik und seit 2016 Geschäftsführer (CTO) der DKMS Life Science Lab GmbH in Dresden.


Literatur

[1] Zhu N Zhang D Wang W A novel coronavirus from patients with pneumonia in China, 2019 N Engl J Med 2020 382 727 733 10.1056/NEJMoa2001017 31978945 PMC7092803
[2] Lange V Böhme I Hofmann J Cost-efficient high-throughput HLA typing by MiSeq amplicon sequencing BMC Genom 2014 15 63 10.1186/1471-2164-15-63 PMC3909933 24460756
[3] Klussmeier A Massalski C Putke K High-throughput MICA/B genotyping of over two million samples: workflow and allele frequencies Front Immunol 2020 11 314 10.3389/fimmu.2020.00314 32153595 PMC7047279
[4] Corman VM Landt O Kaiser M Detection of 2019 novel coronavirus (2019-nCoV) by real-time RT-PCR Eurosurveillance 2020 25 2000045 10.2807/1560-7917.ES.2020.25.3.2000045 PMC6988269 31992387
[5] van Kasteren PB van der Veer B van den Brink S Comparison of seven commercial RT-PCR diagnostic kits for COVID-19 J Clin Virol 2020 128 104412 10.1016/j.jcv.2020.104412 32416600 PMC7206434
[6] Kleiboeker S Cowden S Grantham J SARS-CoV-2 viral load assessment in respiratory samples J Clin Virol 2020 129 104439 10.1016/j.jcv.2020.104439 32674034 PMC7235577
[7] Pan Y Zhang D Yang P Viral load of SARS-CoV-2 in clinical samples Lancet Infect Dis 2020 20 411 412 10.1016/S1473-3099(20)30113-4 32105638 PMC7128099
[8] Deckert A, Bärnighausen T, Kyei N (2020) Pooled-sample analysis strategies for COVID-19 mass testing: a simulation study. Bull World Health Organ, 10.2471/BLT.20.25805310.2471/BLT.20.257188 PMC7463190 33012859
